# Effect of heat stress in the first 1000 days of life on fetal and infant growth: a secondary analysis of the ENID randomised controlled trial

**DOI:** 10.1016/S2542-5196(24)00208-0

**Published:** 2024-10-08

**Authors:** Ana Bonell, Ana M Vicedo-Cabrera, Giovenale Moirano, Bakary Sonko, David Jeffries, Sophie E Moore, Andy Haines, Andrew M Prentice, Kris A Murray

**Affiliations:** a Medical Research Council Unit The Gambia at London School of Hygiene & Tropical Medicine, Fajara, The Gambia; b Centre on Climate Change and Planetary Health, London School of Hygiene & Tropical Medicine, London, UK; c Institute of Social and Preventive Medicine and Oeschger Center for Climate Change Research, University of Bern, Bern, Switzerland; d Department of Medical Sciences, University of Turin, Turin, Italy; e Department of Women and Children's Health, King's College London, London, UK

## Abstract

**Background:**

The intersecting crises of climate change, food insecurity, and undernutrition disproportionately affect children. Understanding the effect of heat on growth from conception to 2 years of age is important because of mortality and morbidity implications in the near term and over the life course.

**Methods:**

In this secondary analysis, we used longitudinal pregnancy cohort data from the Early Nutrition and Immunity Development (ENID) randomised controlled trial in West Kiang, The Gambia, which occurred between Jan 20, 2010, and Feb 10, 2015. The ENID trial assessed micronutrient supplementation in the first 1000 days of life starting from 20 weeks’ gestation, during which anthropometric measurements were collected prospectively. We used multivariable linear regression to assess the effect of heat stress (defined by Universal Thermal Climate Index [UTCI]) on intrauterine growth restriction based on length-for-gestational age Z score (LGAZ), weight-for-gestational age Z score (WGAZ), and head circumference-for-gestational age Z score (HCGAZ) at birth, and assessed for effect modification of supplement intervention on the relationship between heat stress and infant anthropometry. We used multivariable, multilevel linear regression to evaluate the effect of heat stress on infant growth postnatally based on weight-for-height Z score (WHZ), weight-for-age Z score (WAZ), and height-for-age Z score (HAZ) from 0 to 2 years of age.

**Findings:**

Complete data were available for 668 livebirth outcomes (329 [49%] female infants and 339 [51%] male infants). With each 1°C increase in mean daily maximum UTCI exposure, in the first trimester, we observed a reduction in WGAZ (–0·04 [95% CI –0·09 to 0·00]), whereas in the third trimester, we observed an increase in HCGAZ (0·06 [95% CI 0·00 to 0·12]), although 95% CIs included 0. Maternal protein-energy supplementation in the third trimester was associated with reduced WGAZ (–0·16 [–0·30 to –0·02]) with each 1°C increase in mean daily maximum UTCI exposure, while no effect of heat stress on WGAZ was found with either standard care (iron and folate) or multiple micronutrient supplementation. For the postnatal analysis, complete anthropometric data at 2 years were available for 645 infants (316 [49%] female infants and 329 [51%] male infants). Postnatally, heat stress effect varied by infant age, with infants aged 6–18 months being the most affected. In infants aged 12 months exposed to a mean daily UTCI of 30°C (preceding 90-day period) versus 25°C UTCI, we observed reductions in mean WHZ (–0·43 [95% CI –0·57 to –0·29]) and mean WAZ (–0·35 [95% CI –0·45 to –0·26]). We observed a marginal increase in HAZ with increasing heat stress exposure at age 6 months, but no effect at older ages.

**Interpretation:**

Our results suggest that heat stress impacts prenatal and postnatal growth up to 2 years of age but sensitivity might vary by age. In the context of a rapidly warming planet, these findings could have short-term and long-term health effects for the individual, and immediate and future implications for public child health.

**Funding:**

Wellcome Trust.

## Introduction

With anthropogenic climate change having already heated the planet by a mean average of 1·2°C above pre-industrial temperatures, interest is growing in the effect of ambient temperatures on both fetal and infant health.^1^, ^2^ The current intersecting crises of climate change, crop failure, extreme heat, drought, and globally rising food costs disproportionately affect children living in extreme poverty.^3^ Although the impact of extreme heat on pregnancy outcomes has been explored in multiple studies,^4^, ^5^ longitudinal studies on child growth are scarce. Growth faltering, defined as a slower rate of weight or height gain in childhood than expected for age and sex, is known to be multifactorial. It is influenced by genetic and epigenetic variants, food insecurity, poverty, poor sanitation, repeated or chronic infections, the gut microbiota, emotional and physical neglect, and environmental exposures.^6^, ^7^, ^8^ Recent studies have suggested that heat stress (defined as the net heat load, including from air temperature, humidity, solar radiation, wind speed, metabolic heat production, and clothing insulation) might be one of these environmental factors.^9^, ^10^, ^11^ For example, Tusting and colleagues found an increased prevalence of wasted (weight-for-height Z score [WHZ] <–2 SD) and underweight (weight-for-age Z score [WAZ] <–2 SD) children in those exposed to temperatures higher than 35°C.^11^ Children younger than 2 years were more vulnerable to the effects of heat versus those aged 2–5 years, suggesting a possible increased susceptibility to heat stress in early life.^11^ However, the cross-sectional design of studies to date^9^, ^10^, ^11^ makes interpretation of these findings difficult, as growth faltering occurs over time. A further key knowledge gap exists regarding the interaction between nutritional supplementation, heat stress, and growth faltering, both in pregnancy and infancy. For example, Shankar and colleagues found that maternal heat exposure in the first trimester was associated with poor fetal growth; however, this association disappeared in women receiving micronutrient supplementation in the 3 months before conception.^12^

**Research in context**

**Evidence before this study**

A growing body of evidence is demonstrating the harm of climate change on fetal and child health. We searched PubMed and Ovid from database inception up to July 1, 2024, for evidence of the effect of heat or extreme heat on both fetal and infant growth, using the terms “heat” or “heat stress” or “extreme heat” and “small for gestational age” or “low birth weight” or “intrauterine growth restriction” or “growth faltering” or “wasting” or “stunting” or “underweight”, with search results restricted to publications in English. Most studies showed a negative association between maternal or infant heat exposure and neonatal or infant anthropometric measurements. There were more studies exploring the link between heat exposure and low birthweight than any of the other growth outcomes. The effect of heat exposure on infant growth has largely been explored in large cross-sectional studies, from which evidence suggests that heat exposure increases the risk of wasting. Evidence has been conflicting on the effect of heat on stunting in early life.

**Added value of this study**

Our study fills an important research gap by using longitudinal data to track growth in a birth cohort during the first 1000 days of life, assessing the negative effects of heat stress exposure and indicating at-risk windows. We found that maternal exposure to heat stress in the first trimester was associated with a reduction in weight-for-gestational age Z score at birth. In addition, weight-for-height Z scores and weight-for-age Z scores were reduced in infants aged up to 2 years with increasing heat stress exposure in the preceding 3 months.

**Implications of all the available evidence**

The findings of this study indicate that environmental heat exposure is associated with negative growth both in utero and in infancy. With global rates of child wasting remaining unacceptably high and ongoing planetary warming, these findings should spur action on improving child health.

**Figure 1:**
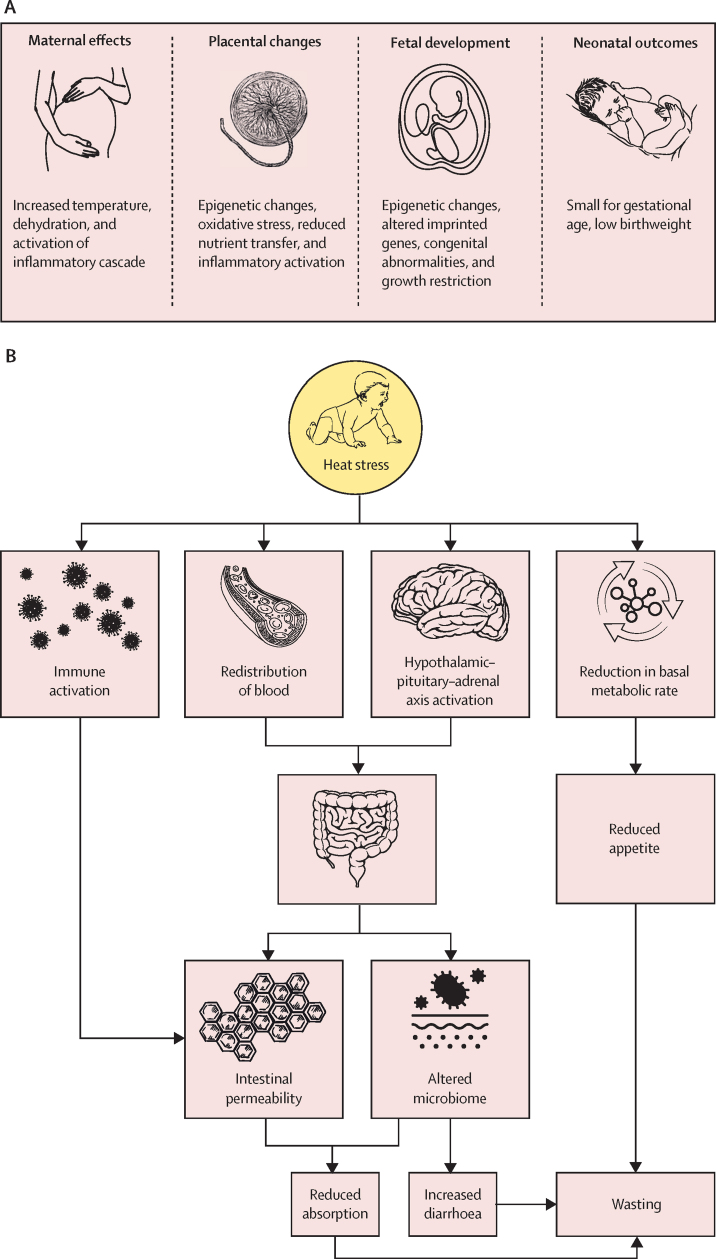
Schematic of potential biological pathways of heat stress effects on growth in-utero (A) and in infancy (B)

Several mechanisms might explain the possible link between heat exposure and growth faltering, with potentially distinct pathways acting prenatally and postnatally (figure 1A, B). In infants and young children, the possible mechanisms to explain how heat stress increases the risk of wasting and underweight include: reduction in food intake to reduce metabolic heat production and so reduce thermal gain;^13^ activation of the hypothalamic–pituitary–adrenal axis;^14^ alterations in the microbiome due to heat stress and poor food storage before eating (increased microbial growth), leading to increased risk of acute episodes of diarrhoea;^15^ immune activation leading to reduced nutritional absorption;^16^ and near-term household food insecurity due to heat and drought affecting crop yield (figure 1B).^17^ The pathways involved in stunting (height-for-age Z score [HAZ] <–2 SD) and heat stress are more complex than wasting and likely involve several indirect pathways such as reduced crop yields leading to long-term food insecurity, reduced water access, and low resilience to acute weather events within the health system affecting vaccination campaigns, access to health care, and access to antenatal services.^10^, ^18^

West Africa is highly vulnerable to the effects of climate change, has experienced an increase in the duration, frequency, and intensity of heat waves in the past 10 years, and has one of the highest rates of undernutrition globally.^19^, ^20^ Studies have shown limited benefits of nutrition interventions and no effect from water, sanitation, and hygiene interventions in reducing growth faltering.^21^, ^22^ These outcomes are concerning as wasting and stunting have short-term and long-term health implications and are associated with increased mortality, reduced immunity, developmental delays, a life-course increased risk of chronic health conditions such as type 2 diabetes, and reduced economic productivity in adulthood.^23^, ^24^, ^25^ As climate change is a threat multiplier of health vulnerabilities, and UNICEF has declared that the climate crisis is a child rights crisis,^26^ it is of growing importance to understand the effect of heat stress on growth faltering in West Africa, identify vulnerable age groups, and understand the potential interaction of nutritional supplementation.

In the present study, we used longitudinal pregnancy cohort data from the Early Nutrition and Immunity Development (ENID) randomised trial, which assessed nutritional supplementation in the first 1000 days of life (the time from conception to 2 years of age), during which multiple repeated measures were collected prospectively in individual pregnant women and their children. With these data, we examined the relationship between ambient heat stress exposure and growth faltering using multivariate and multilevel linear models incorporating maternal, child, and environmental factors, and we explored at-risk age windows of exposure, and the interaction with maternal nutritional supplementation.

## Methods

### Pregnancy cohort data

The ENID randomised trial was based in West Kiang, The Gambia, from Jan 20, 2010, to Feb 10, 2015. This rural, resource-poor region has seasonal variations in climate, nutritional exposures, and birth outcomes.^27^, ^28^ The ENID trial recruited pregnant women (aged 17–47 years) from the entire region if they consented and fulfilled the following criteria: confirmed pregnancy of less than 20 weeks’ gestation (singleton pregnancy), not in another UK Medical Research Council (MRC) study, haemoglobin higher than 7 g/dL, and had not self-reported the onset of menopause. Obstetric ultrasound was used to determine gestational age at entry into the trial (mean gestational age at enrolment 13·3 weeks [SD 3·37]), via crown-rump length (if <14 weeks’ gestation) or biparietal diameter (if ≥14 and <20 weeks’ gestation). The trial aimed for complete data on 800 mother–infant pairs. Full details of the trial are in the published protocol.^29^

Pregnant women underwent masked random allocation into one of four groups, in which they received one of the following daily supplementations from 20 weeks’ gestation until delivery: (1) control (standard care; iron and folate supplements); (2) multiple micronutrients; (3) protein-energy; or (4) multiple micronutrients and protein-energy. Participants in groups 2–4 also received daily iron and folate supplements from 20 weeks’ gestation until delivery. In addition, infants between 6 and 18 months of age underwent masked random allocation to two further daily nutritional supplement groups: (1) lipid-based supplement alone; or (2) lipid-based supplement with micronutrients. Full details of the supplements are provided in the protocol.^29^ Mother–infant pairs were visited within 72 h of delivery for a health assessment and collection of newborn anthropometries and birth data when possible.^30^ Due to the time-sensitive nature of this visit, 668 infants had complete birth data. Length-for-gestational age Z score (LGAZ), weight-for-gestational age Z score (WGAZ), and head circumference-for-gestational age Z score (HCGAZ) were calculated with the INTERGROWTH-21st standards.^30^ After birth, weight and length measurements were collected at the following weeks of age: 1, 8, 12, 24, 40, 52, 78, and 104.^31^ Postnatal WAZ, HAZ, and WHZ from 1 month to 2 years were calculated with the WHO Multicentre Growth Reference Study growth curves.^32^ Once-weekly visits to participants collected questionnaire data on any acute illness, including fever, diarrhoea, vomiting, cough, difficulty in breathing, or any other infection in the preceding week. Complete data were available for 645 infants from 1 month to 2 years.

Ethics approval for the original study was granted by The Gambia Government/MRC Laboratories Joint Ethics Committee (Banjul, The Gambia) in August, 2008 (reference SCC 1126v2) and further ethical approval was obtained in April, 2021, from The Gambia Government/MRC Joint Ethics Committee and the London School of Hygiene & Tropical Medicine Ethics Advisory Board (London, UK; reference 25171) for the present analysis.

### Environmental exposure data

**Figure 2:**
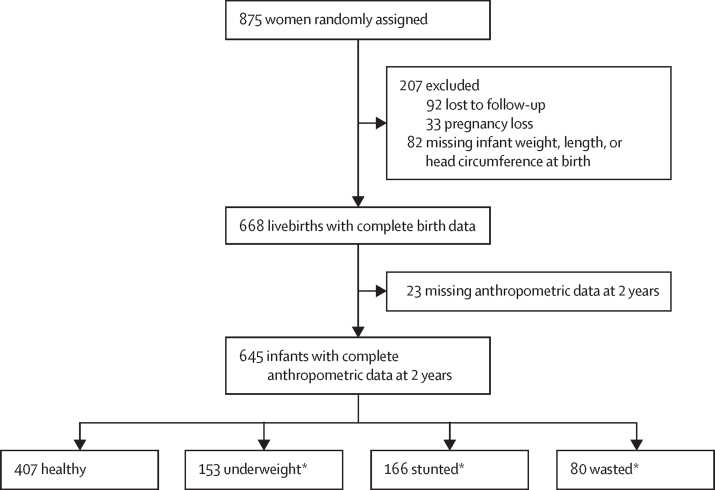
Flowchart of populations included in analysis Underweight: <–2 weight-for-age Z score; stunted: <–2 height-for-age Z score; wasted: <–2 weight-for-height Z score. *Individuals could be included in more than one group.

The ERA5-HEAT (Human Thermal Comfort) dataset from the Copernicus Climate Change Service was used to establish location-specific heat stress.^33^ ERA5 uses quality-controlled historical meteorological station observations to give global gridded estimates by advanced modelling and data assimilation. Data are gridded at 0·25 by 0·25 degrees, and are available hourly. We used the Universal Thermal Climate Index (UTCI; a composite measure of heat, humidity, wind speed, and solar radiation) as the heat stress index, due to variation in humidity by season in The Gambia and because UTCI has been shown to more effectively capture the physiological response to heat stress exposure than air temperature alone, including in previous studies of heat stress in pregnancy in The Gambia.^34^, ^35^, ^36^, ^37^ Hourly data were summarised to daily maximum, mean, minimum, and diurnal UTCI and matched to mother–infant pairs by geolocation of village of residence (figure 2).

### Statistical analysis

Descriptive data are expressed as mean and SD. Z scores outside of biological plausibility (>4 or <–4) were removed and individuals with missing data were excluded for analyses. Thus, the in-utero analysis included all individuals with complete birth data, and the infant analysis included all individuals with complete anthropometric data at 2 years. There is uncertainty in the global consensus on definitions of heat stress exposure and health outcomes. For example, heat stress defined by mean daily values, diurnal variation, mean monthly values, maximum monthly values, and minimum monthly values have all been shown to be associated with poor pregnancy and infant outcomes.^4^, ^5^, ^10^, ^11^ Due to the different biological and physiological pathways and timespans involved in wasting and stunting, the same exposure definition for heat stress by condition was considered to be highly unlikely. Therefore, we did not a-priori assume a best-fit exposure definition for heat stress, nor did we apply the same definition of heat stress to the different outcomes.

To analyse the effects of heat stress in-utero, linear regression models with village as a cluster variable were run on a-priori defined covariables. These included maternal factors (age, education, and mid-upper arm circumference), infant sex, intervention group (third trimester only), season of conception (modelled with cubic splines with 3 degrees of freedom), and parity. Heat stress defined as daily minimum, mean, maximum, or diurnal exposure, mean averaged by trimester, was assessed with use of the Akaike information criterion, with the lowest-value model taken as best fit, which was the mean daily maximum UTCI per trimester. Thus, we assessed the change in growth metrics (LGAZ, WGAZ, and HCGAZ) per 1°C increase in mean daily maximum UTCI exposure by trimester. Trimesters were defined as first trimester (conception to 12 weeks and 6 days of gestation), second trimester (13 weeks to 27 weeks and 6 days of gestation), and third trimester (28 weeks of gestation and onwards). As it has been proposed that nutritional supplementation might modify the effect of extreme heat in pregnancy,^12^ this was assessed by evaluating maternal supplementation as an effect modifier. The original study design evaluated control supplementation versus the two interventions during pregnancy, with anyone who received multiple micronutrients grouped together (including individuals who received both multiple micronutrients and protein-energy), and anyone who received protein-energy grouped together (also including the individuals who received both multiple micronutrients and protein-energy).^29^ We followed the same design and thus analysed three groups. Given that supplementation was started at 20 weeks’ gestation, the interaction of supplementation on the effect of heat stress on growth was only evaluated in the third trimester. Additionally, we assessed the effect of supplementation in the third trimester in a sensitivity analysis, in which extreme heat exposure was defined according to a previous published study,^12^ as daily maximum temperatures exceeding 39°C for 20 days or more. The mean difference in birth anthropometric measurements in newborn infants exposed to extreme heat in the third trimester versus those not exposed, by supplementation group, was evaluated with the Wilcoxon rank-sum test (significance level p<0·05).

To evaluate the relationship between postnatal exposure to heat stress and infant growth, mixed-effect linear regressions were used, with random effects for infants within each village (as a cluster variable). A quadratic correlation structure was fitted to the repeated anthropometric measurements (HAZ, WHZ, and WAZ), which allowed for the intrasubject correlation. The effects of seasonality and time were modelled using natural cubic splines with 11 degrees of freedom and three knots (allowing a monthly covariate), with the non-linear effects of age modelled using a cubic spline with three degrees of freedom and two knots, and the heat index modelled using a cubic spline with two degrees of freedom and one knot. As with the in-utero model, daily mean, minimum, maximum, and diurnal heat stress exposure were considered. These exposures were mean averaged over 30-day retrospective windows (constrained by infant age) of 30 to 360 days (ie, from 1 month exposure up to 1 year exposure before the measurement), as well as a mean lifetime exposure. A grid search method for each of HAZ, WHZ, and WAZ was used to identify the best spline orders, the most appropriate heat index with retrospective window size, and to select predefined covariates (from maternal and infant supplementation, birthweight, maternal BMI or maternal height, gestational age at birth, infant sex, maternal age at birth, parity, maternal education, exclusively breastfed, infant illness in the preceding 4 weeks before growth measurement, and mean daily UTCI from conception to delivery), with an evaluation of effect modification by infant age. The final model was selected as the model with minimum Akaike information criterion value with all covariates significant at the 5% level (appendix pp 5–6).^38^ Standard residual diagnostics were used for the chosen regression models and a semi-variogram for the within-subject correlation structure. Of note, this method is in accordance with hypothesis generating, rather than hypothesis testing.

All statistical analyses were done in R (version 4.1.0). Confidence intervals were calculated for all the regression models in R. We present confidence intervals to allow interpretation of findings without presentingp values.

### Role of the funding source

The funder of the study had no role in study design, data collection, data analysis, data interpretation, or writing of the report.

## Results

Basic maternal sociodemographic characteristics are presented in table 1. Complete data were available for 668 livebirth outcomes (329 [49%] female infants and 339 [51%] male infants), with a mean birthweight of 3·00 kg (SD 0·40). At birth, 66 (10%) infants weighed less than 2·5 kg (low birthweight), 218 (33%) were small for gestational age (defined as <10th percentile of weight for gestational age),^39^, ^40^ and nine (1%) were preterm (<37 weeks’ gestation). For the infant analysis, complete anthropometric data at 2 years were available for 645 infants (316 [49%] female infants and 329 [51%] male infants; figure 2). Mean WHZ was –0·94 (SD 0·98), mean WAZ was –1·31 (0·96), and mean HAZ was –1·26 (0·99). At any timepoint during the study period, 121 (19%) of 645 infants were wasted, 183 (28%) were underweight, and 185 (29%) were stunted.

**Table 1:** Maternal sociodemographic characteristics at enrolment

	**Mean (SD) or median (IQR)**
Age, years	29·7 (6·6)
Height, cm	162·0 (5·8)
BMI, kg/m^2^	21·1 (5·8)
Mid-upper arm circumference, cm	26·6 (3·1)
Time in education, years	1·4 (3·0)
Parity	4 (2–6)

**Figure 3:**
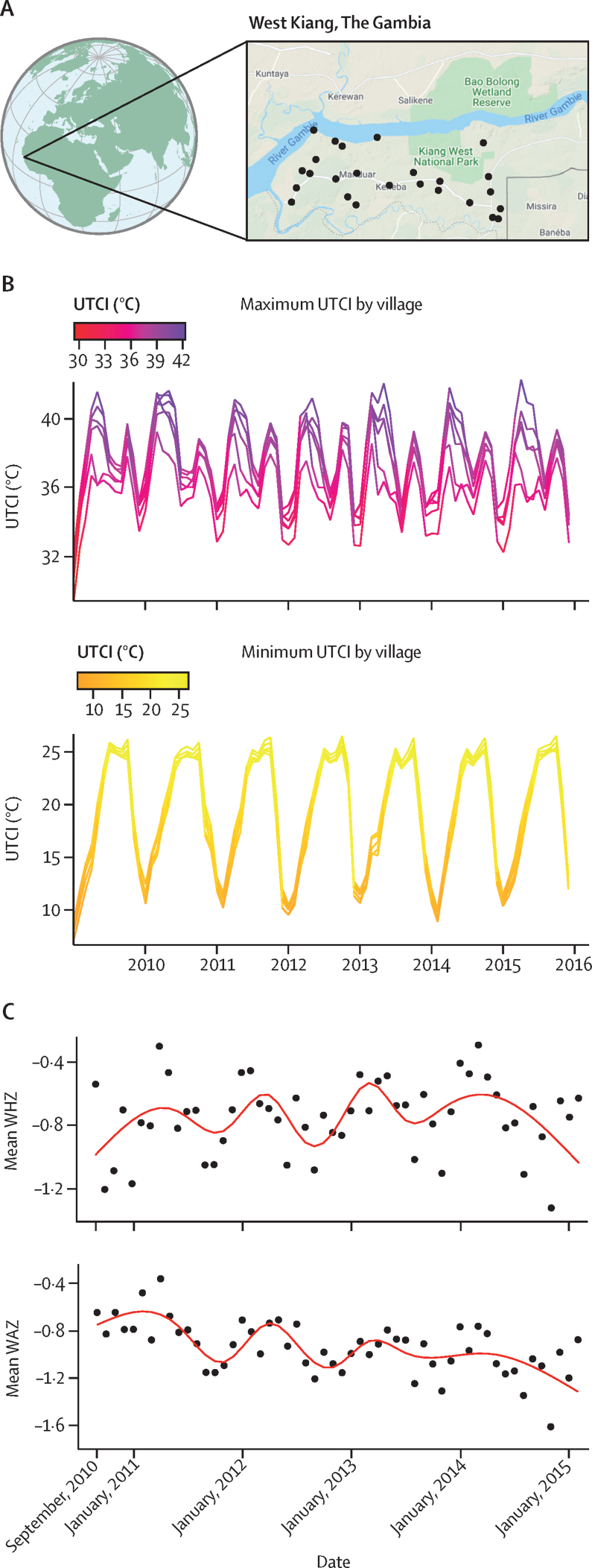
Geographic region (A), heat stress exposure over the course of the study (B), and seasonal trends in WHZ and WAZ (C) In panel A, black dots represent village locations. Map data ©2021 Google. In panel B, each line is an individual village exposure. UTCI=Universal Thermal Climate Index. WAZ=weight-for-age Z score. WHZ=weight-for-height Z score.

Daily mean UTCI exposure over the study period ranged from 11·6°C to 34·0°C. Averaged over all study days, the mean daily UTCI exposure was 26·9°C (SD 3·8). The highest daily maximum UTCI was 45·7°C and highest daily minimum was 28·9°C. Figure 3 displays the study region, the seasonal nature of heat stress exposure by village, and the seasonal trend in both WHZ and WAZ. More detailed plots of the modelled seasonality are provided in the appendix (pp 3–4).

The in-utero model showed a decrease in WGAZ (–0·04 [95% CI –0·09 to 0·00]; although the upper 95% CI was 0) with each 1°C increase in mean daily maximum UTCI exposure in the first trimester (table 2). Heat stress exposure in the second trimester had no effect on intrauterine growth (with all 95% CIs crossing the null). In the third trimester, there was an increase in HCGAZ (0·06 [95% CI 0·00 to 0·12]; although the lower 95% CI was 0) with each 1°C increase in mean daily maximum UTCI exposure. We did not find evidence of an effect of heat stress on LGAZ in any trimester.

**Table 2:** Associations between mean daily maximum exposure to heat stress (per 1°C increase in UTCI) in pregnancy and newborn anthropometric measurements

	Estimate (95% CI)*
**LGAZ**	
First trimester	−0·03 (−0·09 to 0·02)
Second trimester	0·00 (−0·06 to 0·06)
Third trimester	0·03 (−0·03 to 0·09)
**HCGAZ**	
First trimester	−0·04 (−0·10 to 0·01)
Second trimester	0·02 (−0·04 to 0·08)
Third trimester	0·06 (0·00 to 0·12)
**WGAZ**	
First trimester	−0·04 (−0·09 to 0·00)
Second trimester	0·03 (−0·03 to 0·08)
Third trimester	0·07 (−0·03 to 0·18)
**WGAZ by supplement group in the third trimester**†	
Control supplementation	−0·27 (−0·11 to 0·01)
Multiple micronutrient supplementation	−0·08 (−0·20 to 0·04)
Protein-energy supplementation	−0·16 (−0·30 to −0·02)

Trimesters were defined as: first trimester=conception to 12 weeks and 6 days of gestation; second trimester=13 weeks to 27 weeks and 6 days of gestation; and third trimester=28 weeks of gestation and onwards. HCGAZ=head circumference-for-gestational age Z score. LGAZ=length-for-gestational age Z score. UTCI=Universal Thermal Climate Index. WGAZ=weight-for-gestational age Z score.

* Beta coefficients for a 1°C increase in the mean daily maximum UTCI per trimester, adjusted for maternal age, infant sex, season of conception, maternal mid-upper arm circumference, parity, maternal education, village of residence (cluster variable), and intervention group (in the third trimester only).

† Including an interaction term for UTCI by supplement group in the third trimester.

Nutritional supplementation in the third trimester modified the effect of UTCI on WGAZ. WGAZ was reduced (–0·16 [95% CI –0·30 to –0·02]) for each 1°C increase in mean daily maximum UTCI exposure in the protein-energy supplementation group, with no effect of heat stress in either the standard care or multiple micronutrient supplementation groups (table 2). There was no supplementation effect modification on LGAZ and HCGAZ (data not shown).

Associations were further assessed in a sensitivity analysis in which extreme heat stress was defined as 20 days or more of a mean daily maximum UTCI of 39°C or higher in the third trimester. We found no evidence of differences in mean birth anthropometric measurements in those exposed to extreme heat stress in the third trimester versus those unexposed in both the control supplementation and multiple micronutrient supplementation groups. However, mothers in the protein-energy supplementation group who were exposed to extreme heat had newborn infants with a significantly lower mean WGAZ than those who were unexposed to extreme heat (appendix p 2).

**Figure 4:**
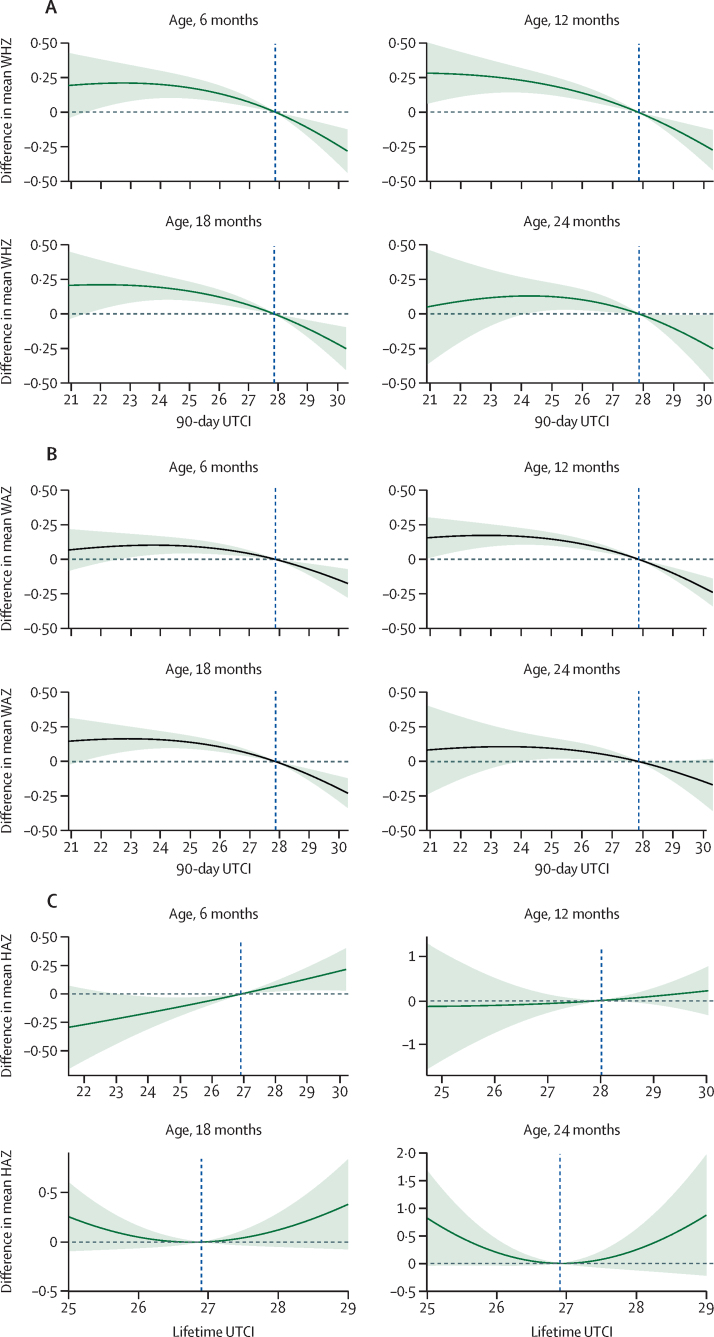
Association between infant anthropometry and UTCI (A) Association between WHZ and mean daily UTCI for the preceding 90-day period, adjusted for season, birthweight, gestational age at birth, infant sex, maternal BMI, and maternal age. (B) Association between WAZ and 90-day mean daily UTCI, adjusted for season, birthweight, infant sex, and parity. (C) Association between HAZ and mean daily lifetime UTCI, adjusted for season, birthweight, gestational age at birth, maternal height, infant sex, and parity. The relationship between heat and growth varied by age and therefore age intervals are presented separately. The solid line represents the mean Z score estimates and the shading represents 95% CIs. The vertical dashed line is median exposure for the cohort. HAZ=height-for-age Z score. UTCI=Universal Thermal Climate Index. WAZ=weight-for-age Z score. WHZ=weight-for-height Z score.

For postnatal heat stress exposure, exposure–response associations varied by age for WHZ, WAZ, and HAZ (figure 4; full model outputs in the appendix [pp 6–7]). The WHZ and WAZ curves give the estimated Z score for infants exposed to the level of heat stress (mean daily UTCI) over the preceding 90 days, adjusted for co-variables (figure 4A, B). We observed a negative association between the Z scores (WHZ or WAZ) and exposure to increasing UTCI. This negative association was apparent up to 24 months of age with infants aged 6–18 months showing the largest decreases in WHZ and WAZ. For a 12-month-old infant exposed to 30°C UTCI versus 25°C UTCI, we observed reductions in mean WHZ (–0·43 [95% CI –0·57 to –0·29]) and mean WAZ (–0·35 [95% CI –0·45 to –0·26]). For an 18-month-old infant similarly exposed, WHZ changed by –0·38 [–0·52 to 0·24]) and WAZ reduced by –0·34 (–0·43 to –0·24), and for a 24-month-old infant, WHZ reduced by –0·34 (–0·55 to –0·13) and WAZ by –0·24 (–0·40 to –0·08). In the HAZ model, the best definition for heat stress was mean lifetime exposure, which is in keeping with the long timeframe over which stunting occurs^41^ (appendix p 7). For most exposures we found no association between HAZ and heat stress at each age, with the 95% CIs including 0, except for a small range of exposures in the 6-month age group. For a 6-month-old infant with a lifetime exposure of 25°C versus 30°C UTCI, mean HAZ increased by 0·32 (95% CI 0·16 to 0·48).

## Discussion

In this analysis, we found that in-utero exposure to heat stress in the first trimester corresponded with a reduction in WGAZ, and that protein-energy supplementation had a negative effect on the relationship between heat stress and WGAZ in the third trimester. These findings build on previous evidence showing that the first trimester is a vulnerable time to heat exposure, when changes in the in-utero environment can have lifetime effects.^41^ For example, Shankar and colleagues found a similar effect of heat stress on intrauterine growth (LGAZ and HCGAZ) in the first trimester.^12^ Interestingly, they found that micronutrient supplementation before conception modified the effects of heat stress on intrauterine growth in the first trimester and reduced the negative effect of heat stress. This effect and our findings support the growing literature that the biological pathways involved differ by gestational age (figure 1). In utero, the postulated pathways include: first trimester heat stress exposure resulting in abnormal protein folding and subsequent congenital abnormalities,^42^ placental growth suppression,^43^ and variation in fetal DNA methylation and subsequent changes in gene expression;^44^ heat stress exposure at any time during pregnancy resulting in oxidative stress and inflammatory activation causing reduced nutrient transfer;^5^, ^45^ and heat stress exposure in the second and third trimesters resulting in changes in placental blood flow for thermoregulation, leading to acute or chronic fetal stress.^37^ Our findings support the theory that an increased intake of protein-energy supplementation in later pregnancy increases the internal thermal load and hence overall heat stress exposure, which can then be detrimental to fetal growth. However, the increase in HCGAZ associated with heat stress in the third trimester, although in keeping with other studies,^46^ indicates that this relationship is complicated. Therefore, the implications of these findings for wider policy or intervention adoption are unclear and further research into this area is needed.

For infants, we found that increasing heat stress exposure was associated with reductions in WHZ and WAZ after controlling for seasonal trends and maternal and child factors, while there was no effect on HAZ. WHZ showed the largest reductions due to heat stress, and 6–18 months was the most vulnerable age. This finding is concerning as wasting has both short-term and long-term health implications and is associated with increased mortality, reduced immunity, and developmental delays.^23^, ^24^ In addition, although wasting has decreased worldwide over the past two decades, it remains higher than the Sustainable Development Goal target of below 5% globally.^47^ Indeed, in the present cohort, 121 (19%) of 645 infants aged 0–2 years had wasting on at least one occasion. This high rate is consistent with the findings from recent key publications on child wasting and stunting in low-income and middle-income countries.^48^, ^49^, ^50^ These studies also found that WHZ varied in diverse cohorts exposed to seasonal rainfall, with the lowest mean WHZ occurring in the rainiest months. Interestingly, maternal heat exposure during pregnancy was not included in the final best-fitting infant models, implying that there is not a longer-term effect of in-utero heat exposure on the relationship between heat exposure postnatally and infant growth. Our findings mostly corroborate existing literature in this area, although our data sources and methodology differ. For example, previous studies have utilised nutrition indicator data from the Demographic and Health Surveillance programme to explore the relationship between average heat stress exposure and child growth faltering.^9^, ^10^, ^11^ Two of the studies found an increase in child wasting with increasing temperature exposure,^9^, ^11^ as we have shown. Tusting and colleagues found decreased odds of stunting in children aged 0–5 years exposed to temperatures above 35°C compared to below 30°C.^11^ Conversely, a study by Blom and colleagues, of infants aged 3–36 months in West Africa, found a decrease in HAZ with lifetime exposure to temperatures above 35°C, using a cross-sectional design.^10^ In a hospital-based study, Xu and colleagues found an increase in acute hospital admission for undernutrition in Brazil with increases in temperature using a case-crossover design.^50^ Our findings on WHZ and WAZ are in agreement with these studies. However, there might be a complex effect of heat stress on stunting as indicated by conflicting results in the literature, and this should be explored further in larger longitudinal studies in which exposure is more varied and the population less homogeneous.

This study builds on previous work by the MRC Unit in The Gambia. An early study from Rowland and colleagues established a link between growth faltering in children aged 3 months to 3 years and gastrointestinal infections.^51^ Although they found both height and weight to be significantly negatively correlated with diarrhoeal disease, which impeded any catch-up growth that was seen when diarrhoea was not present, subsequent work found no improvement in growth faltering despite a statistically significant reduction in diarrhoea rates.^52^ In our study, neither diarrhoea alone nor any infectious episode in the month preceding anthropometric measurements altered the effect of heat stress on growth faltering metrics and were not included in the final models. Since the 1970s there have been multiple nutritional interventions and improvements in clinical care, vaccinations, access to water and sanitation, and general infrastructure. During this time, infant anthropometry has been routinely collected and allows analysis of changes in growth metrics over time. Nabwera and colleagues analysed these trends and found that the proportion of children with stunting or underweight halved from the 1970s to 2000s, but remained high at 30·0% and 22·1%, respectively, with little change in wasting rates.^21^ This analysis concluded that despite intensive nutrition interventions, factors are missing in the established understanding of contributors to growth faltering. We suggest, based on the results reported herein, that increasing environmental heat stress might be one of those factors. To understand the clinical relevance of these findings, we have compared the estimated change in anthropometric measurements in this study to those reported in association with other interventions or risk factors in other studies and contexts. The change we observed in WGAZ with each 1°C increase in mean daily maximum UTCI in the first trimester was of similar magnitude, albeit in the opposite direction, to that found in a study in New Zealand by Chiavaroli and colleagues, which reported a change in WGAZ with each 1-unit increase in maternal BMI of 0·06 (95% CI 0·00 to 0·11).^53^ The association between heat stress and supplementation was also similar in magnitude, although in the opposite direction, to the findings from a systematic review and meta-analysis of the effects of prenatal supplementation with a lipid-based nutrient on WGAZ (mean difference 0·13 [95% CI 0·05 to 0·21] *vs* iron and folate supplementation or standard of care).^54^ In the infant models, the magnitudes of our findings were again similar to other established determinants of WHZ and WAZ. For example, Moore and colleagues evaluated the effect of prolonged episodes of acute diarrhoea (7–13 days *vs* shorter episodes) on WAZ in children in Brazil and found a similar reduction in WAZ (–0·26)^55^ as we found in infants exposed to 30°C UTCI versus 25°C UTCI. Mertens and colleagues’ pooled longitudinal analysis of 21 child cohorts in low-income and middle-income countries also found a similar mean seasonal decrease in WHZ (–0·27 [95% CI –0·13 to –0·24] during the wettest *vs* driest periods of the year)^49^ as we found in heat-exposed infants. Collectively, although these results do not constitute a hard clinical outcome, we see a similar degree of faltering associated with heat stress as for other factors.

Strengths of the study include that in both the in-utero and infant models, we found little differences in model performance between minimum, mean, or maximum UTCI-defined heat stress, although diurnal UTCI variation performed less well (not shown). This is reassuring in interpreting findings within the literature and for future work. Importantly, the best models according to statistical evaluation also conformed with the proposed biological pathways.

The study limitations include that data came from a randomised controlled trial in one area in The Gambia, limiting generalisability, especially in relation to potential heat stress thresholds of effect that might vary across populations. We used mothers’ residences to geolocate mother–infant pairs, but did not consider relocation or movement within the region. Nevertheless, any such movement is likely to have had a minimal effect as daily temperature variation across the region was small (not shown). We also assumed that the ERA5 grid was an accurate estimate of heat exposure despite potential variation across the grid; however, we did compare onsite measurements to gridded data and found similar values for daytime estimates.^37^ A further limitation is in the absence of a gold-standard heat stress index or defined exposure for children. Although the UTCI was specifically designed as a thermophysiological indicator, model assumptions might not apply to a neonate, infant, or child.^34^, ^56^ We also did not have data on dietary practices, maternal infections, or socioeconomic status, which ideally would have been included.

Future studies to explore heat stress and growth faltering in different populations would help to establish risk thresholds by population, which could inform public health measures. Furthermore, exploration of interventions to reduce heat load (through personal or structural building interventions) and the impact that they have on appetite, food intake, food availability, and infant growth would help to elucidate the causal pathways and potentially interactive effects, as well as help in identifying effective, evidence-based solutions for adapting to global heating.

In conclusion, our study suggests that increasing heat stress is associated with both intrauterine growth restriction and growth faltering postnatally up to 2 years of age. With the current global heat trajectory, our findings have major implications for the protection of child health and development within a changing climate.

### Contributors

### Data sharing

The trial data are not open source and require relevant approvals before access can be granted. Anonymised individual data will be made available from the corresponding author on reasonable request and subject to support from investigators of the original trial, relevant approvals (including ethics), and with a signed data access agreement.

## Declaration of interests

We declare no competing interests.
