# Supplementary material for: Effect of heat stress in the first 1000 days of life on fetal and infant growth: a secondary analysis of the ENID randomised controlled trial
Source: Lancet Planet Health. 2024 Oct 8;8(10):e734–43. doi: 10.1016/S2542-5196(24)00208-0 (PMC11462510; doi:10.1016/S2542-5196(24)00208-0)

THE LANCET
Planetary Health

Supplementary appendix

This appendix formed part of the original submission and has been peer reviewed. We post it as supplied by the authors.

Supplement to: Bonell A, Vicedo-Cabrera AM, Moirano G, et al. Effect of heat stress in the first 1000 days of life on fetal and infant growth: a secondary analysis of the ENID randomised controlled trial. *Lancet Planet Health* 2024; **8:** e734–43.

Appendix

Impact of heat stress in the first 1000 days of life on fetal and infant growth.

Ana Bonell, Ana M Vicedo-Cabrera, Giovenale Moirano, David Jeffries, Bakary Sonko, Sophie E Moore, Andy Haines, Andrew M Prentice, Kris A Murray

Figure 1	Association between extreme heat stress exposure in the third trimester and mean length-adjusted-gestational age z-score, weight-adjusted-gestational age z-score and head circumference-adjusted-gestation age z-score by maternal supplementation	2
Figure 2A	Monthly average weight-for-height z-scores measured over time	3
Figure 2B	Monthly average weight-for-age z-scores measured over time.	3
Figure 2C	Monthly average height-for-age z-scores measured over time.	3
Figure 3A	Season and heat modelled for monthly weight-for-height z-scores.	4
Figure 3B	Season and heat modelled for monthly weight-for-age z-scores.	4
Figure 3C	Season and heat modelled for monthly height-for-age z-scores.	4
Figure 4	Sampling structure by age	5
Table 1	Multilevel, multivariable model of the association between heat stress (defined by universal thermal climate index, UTCI) and weight-for-height z-score in infants aged 0-2 years	5
Table 2	Multilevel, multivariable model of the association between heat stress (defined by universal thermal climate index, UTCI) and weight-for-age z-score in infants aged 0-2 years	5
Table 3	Multilevel, multivariable model of the association between heat stress (defined by universal thermal climate index, UTCI) and height-for-age z-score in infants aged 0-2 years	6
Figure 5A	Akaike Information Criterion (AIC) of different heat stress definitions and weight-for-height z-score models	6
Figure 5B	Akaike Information Criterion (AIC) of different heat stress definitions and weight-for-age z-score models	7
Figure 5C	Akaike Information Criterion (AIC) of different heat stress definitions and height-for-age z-score models	7

Figure 1: Association between extreme heat stress exposure in the third trimester and mean length-adjusted-gestational age z-score, weight-adjusted-gestational age z-score and head circumference-adjusted-gestation age z-score by maternal supplementation

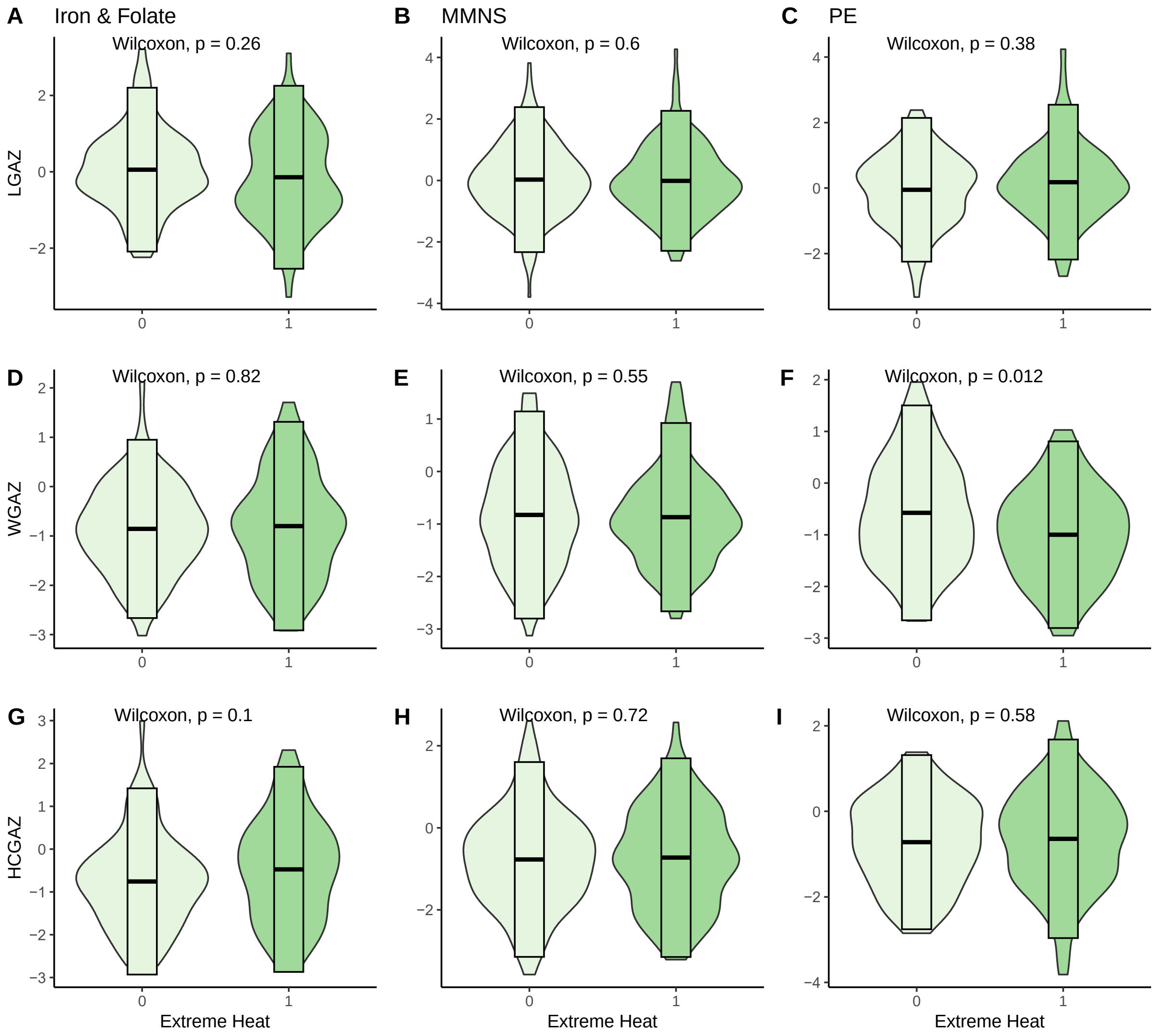

Extreme heat defined as > 20 days with maximum daily UTCI above 39°C throughout the third trimester: 0 = unexposed, 1 = exposed. MMN = micro-nutrient supplementation; PE = protein-energy supplementation; LGAZ = length-adjusted-gestational age z-score; WGAZ = weight-adjusted-gestational age z-score; HCGAZ = head circumference-adjusted-gestational age z-score

Figure 2A: Monthly average weight-for-height z-scores measured over time.

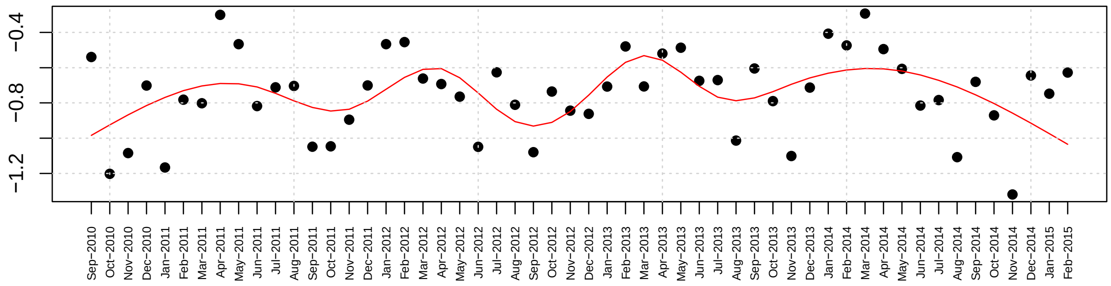

Figure 2B: Monthly average weight-for-age z-scores measured over time

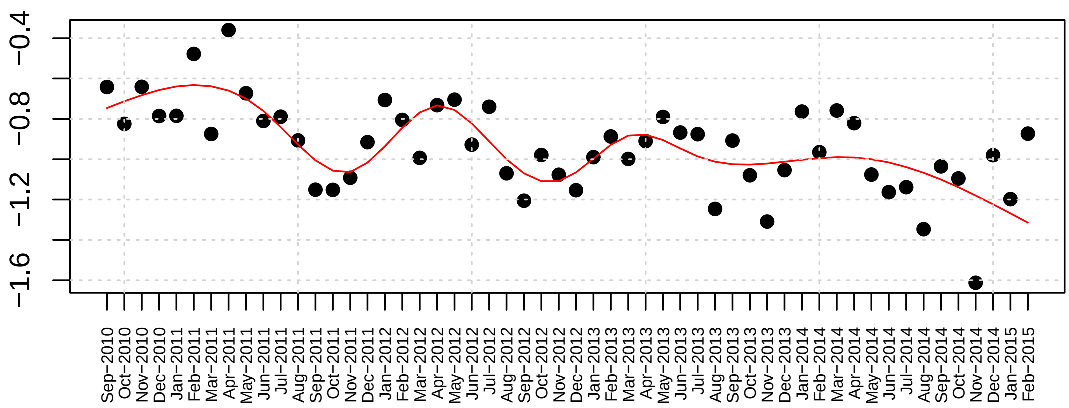

Figure 2C: Monthly average height-for-age z-scores measured over time

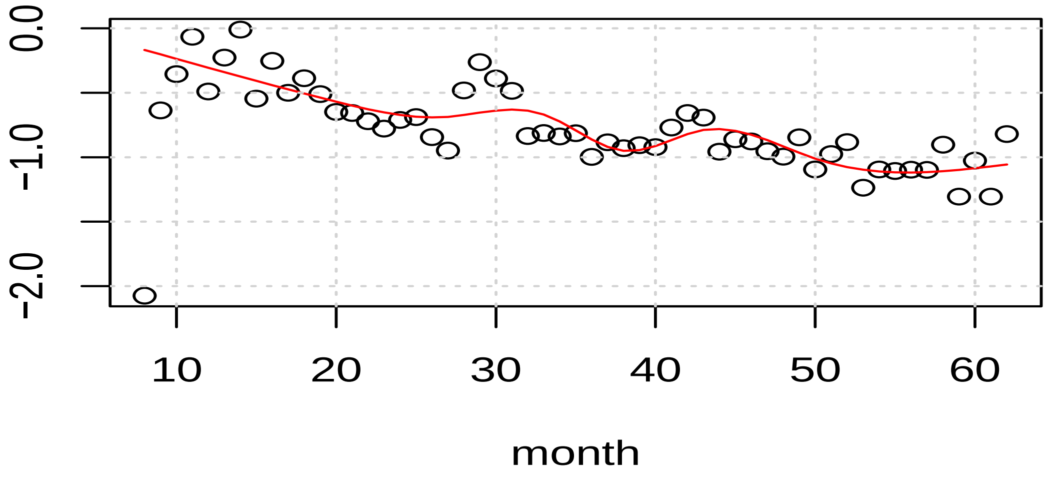

Figure 3A: Season and heat modelled for monthly weight-for-height z-scores.

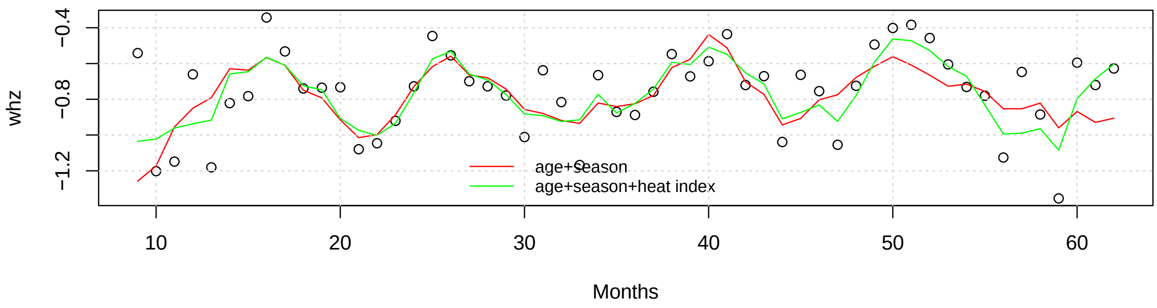

WHZ = Weight-for-height z-score. Red line indicates the model output estimates for mean WHZ when age and season were modelled with splines. Green line indicates the model output estimates for mean WHZ when age, season and heat stress were modelled with splines.

Figure 3B: Season and heat modelled for monthly weight-for-age z-scores.

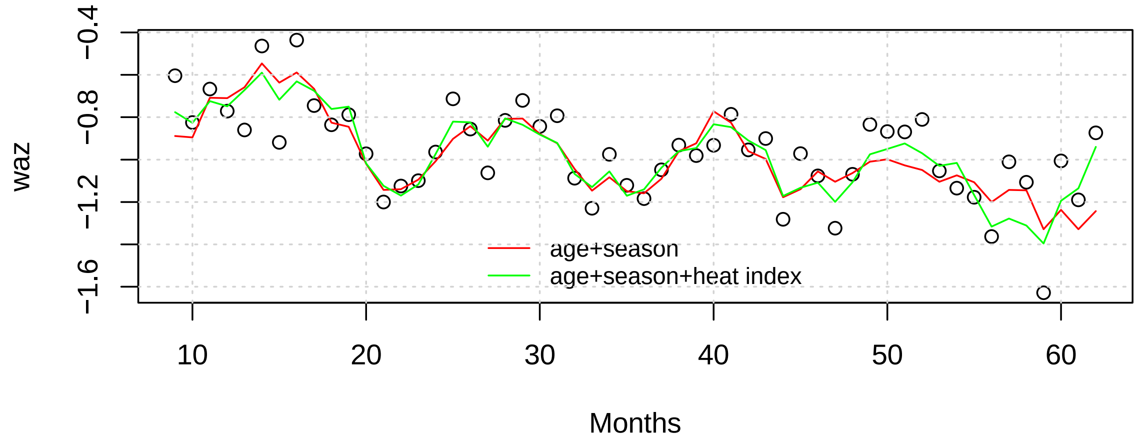

WAZ = Weight-for-age z-score. Red line indicates the model output estimates for mean WAZ when age and season were modelled with splines. Green line indicates the model output estimates for mean WAZ when age, season and heat stress were modelled with splines.

Figure 3C: Season and heat modelled for monthly height-for-age z-scores.

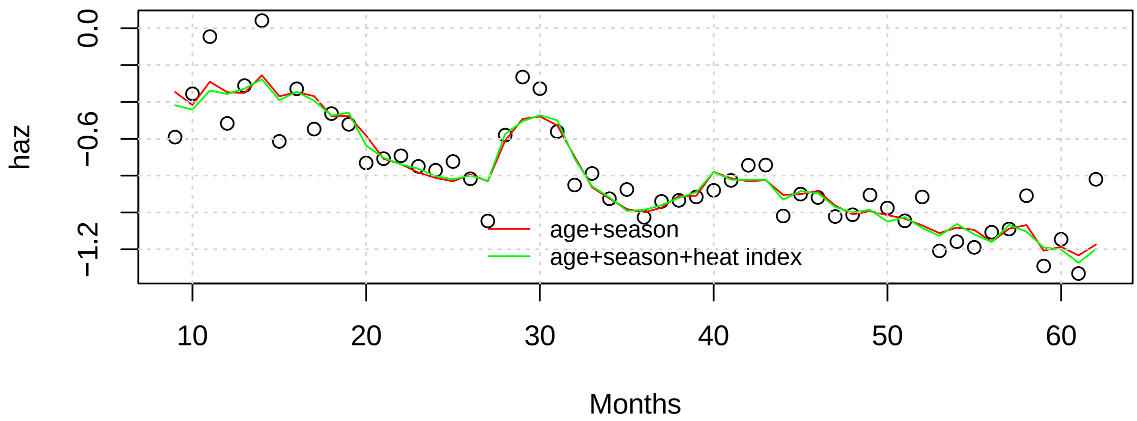

HAZ = Height-for-age z-score. Red line indicates the model output estimates for mean HAZ when age and season were modelled with splines. Green line indicates the model output estimates for mean HAZ when age, season and heat stress were modelled with splines.

Figure 4: Sampling structure by age

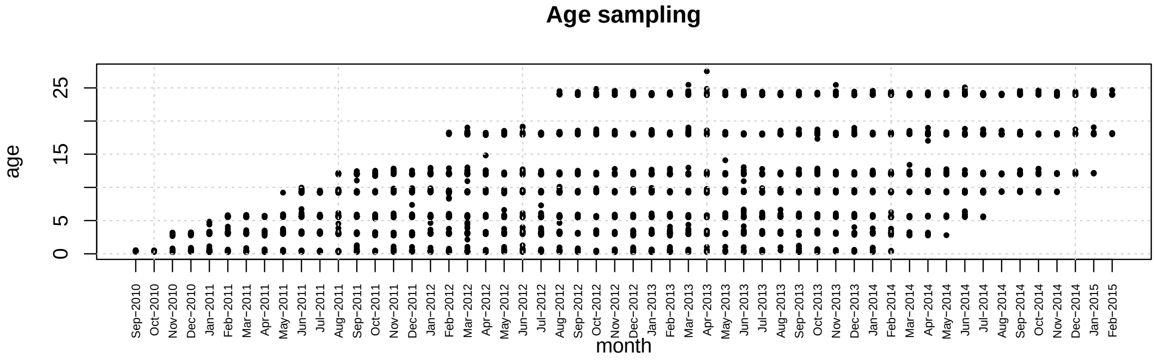

Table 1: Multilevel, multivariable model of the association between heat stress (defined by universal thermal climate index, UTCI) and weight-for-height z-score in infants aged 0-2 years.

Term	Degrees of Freedom	p-value
Season spline	11	<0.001
Age spline	3	0.028
Heat stress spline	2	0.004
Heat spline*Age spline	6	<0.001
Birth weight	1	<0.001
Maternal BMI	1	0.001
Gestational age at birth	1	0.001
Infant sex	1	0.022
Maternal age	1	<0.001

Model presented is the model with the lowest AIC and defined heat stress as average mean UTCI for the preceding 90 days from each measurement. BMI = body mass index

Table 2: Multilevel, multivariable model of the association between heat stress (defined by universal thermal climate index, UTCI) and weight-for-age z-score in infants aged 0-2 years.

Term	Degrees of Freedom	p-value
Season spline	11	<0.001
Age spline	3	0.006
Heat stress spline	2	0.026
Heat spline*Age spline	6	<0.001
Birth weight	1	<0.001
Infant sex	1	<0.001
Parity	1	<0.001

Model presented is the model with the lowest AIC and defined heat stress as average mean UTCI for the preceding 90 days from each measurement.

Table 3: Multilevel, multivariable model of the association between heat stress (defined by universal thermal climate index, UTCI) and height-for-age z-score in infants aged 0-2 years.

Term	Degrees of Freedom	p-value
Season spline	11	<0.001
Age spline	3	0.137
Heat stress spline	2	0.403
Heat spline*Age spline	6	0.003
Birth weight	1	<0.001
Gestational age at birth	1	0.046
Infant sex	1	<0.001
Maternal height	1	<0.001
Parity	1	0.006

Model presented is the model with the lowest AIC and defined heat stress as mean life-time UTCI exposure.

Figure 5A: Akaike Information Criterion (AIC) of different heat stress definitions and weight-for-height z-score models

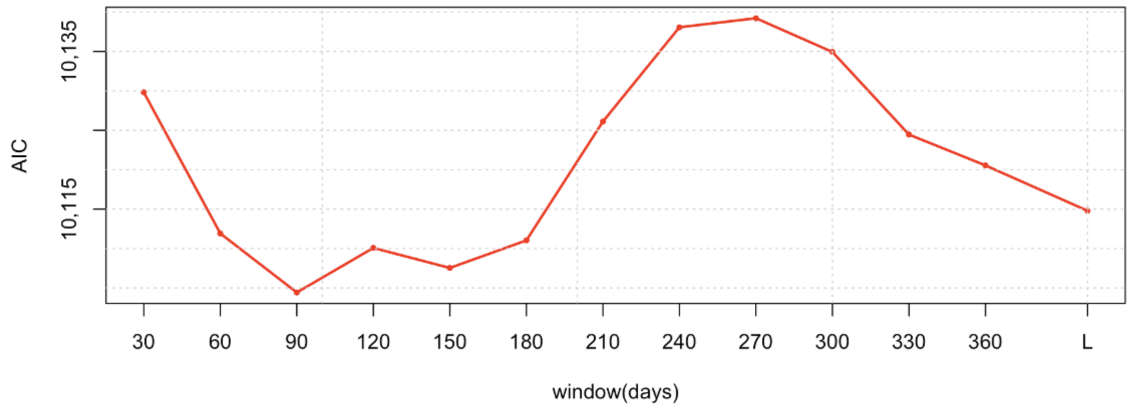

Figure 5B: Akaike Information Criterion (AIC) of different heat stress definitions and weight-for-age z-score models

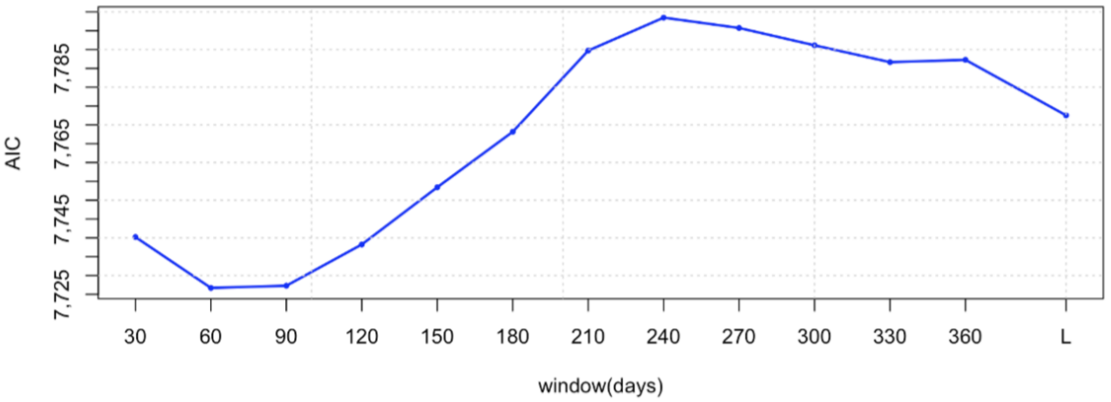

Figure 5C: Akaike Information Criterion (AIC) of different heat stress definitions and height-for-age z-score models

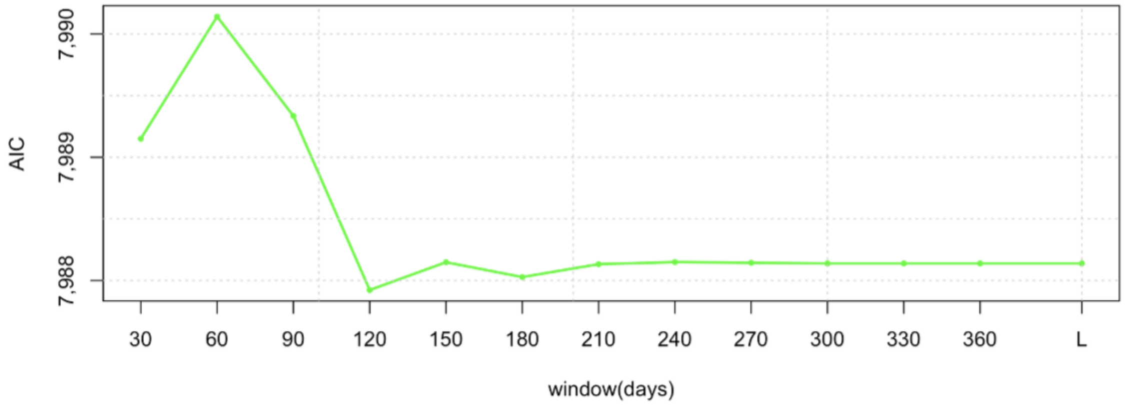

Supplement: Supplementary appendix [file mmc1.pdf]
